# Chronic Kidney Disease among Diabetes Patients in Ethiopia: A Systematic Review and Meta-Analysis

**DOI:** 10.1155/2020/8890331

**Published:** 2020-10-10

**Authors:** Wondimeneh Shibabaw Shiferaw, Tadesse Yirga Akalu, Yared Asmare Aynalem

**Affiliations:** ^1^Department of Nursing, College of Health Science, Debre Berhan University, Debre Berhan, Ethiopia; ^2^Department of Nursing, College of Health Science, Debre Markos University, Debre Markos, Ethiopia

## Abstract

**Background:**

Though different primary studies have reported the burden of chronic kidney disease among diabetes patients, their results have demonstrated substantial variation regarding its prevalence in Ethiopia. Therefore, this study aimed to estimate the pooled prevalence of chronic kidney disease and its associated factors among diabetes patients in Ethiopia.

**Method:**

PubMed, African Journals Online, Google Scholar, Scopus, and Wiley Online Library were searched to identify relevant studies. The *I*^2^ statistic was used to check heterogeneity across the included studies. A random-effects model was applied to estimate the pooled effect size across studies. A funnel plot and Egger's regression test were used to determine the presence of publication bias. All statistical analyses were performed using STATA™ version 14 software.

**Result:**

In this meta-analysis, a total of 12 studies with 4,075 study participants were included. The estimated prevalence of CKD among diabetes patients was found to be 35.52% (95% CI: 25.9–45.45, *I*^2^ = 96.3%) for CKD stages 1 to 5 and 14.5% (95% CI: 10.5–18.49, *I*^2^ = 91.1%) for CKD stages 3 to 5. Age greater than 60 years (OR = 2.99; 95% CI: 1.56–5.73), female sex (OR = 1.68; 95% CI: 1.04–2.69), duration of diabetes >10 years (OR = 2.76; 95% CI: 1.38–5.51), body mass index >30 kg/m^2^ (OR = 2.06; 95% CI: 1.41–3.00), type 2 diabetes (OR = 2.54; 95% CI: 1.73–3.73), poor glycemic control (OR = 2.01; 95% CI: 1.34–3.02), fasting blood glucose >150 mg/dl (OR = 2.58; 95% CI: 1.79–3.72), high density lipoprotein >40 mg/dl (OR = 0.48; 95% CI: 0.30–0.85–25), systolic blood pressure>140 mmHg (OR = 3.26; 95% CI: 2.24–4.74), and diabetic retinopathy (OR = 4.54; CI: 1.08–25) were significantly associated with CKD.

**Conclusion:**

This study revealed that the prevalence of chronic kidney disease remains high among diabetes patients in Ethiopia. This study found that a long duration of diabetes, age>60 years, diabetic retinopathy, female sex, family history of kidney disease, poor glycemic control, systolic blood pressure, overweight, and high level of high-density lipoprotein were associated with chronic kidney disease among diabetic patients. Therefore, situation-based interventions and context-specific preventive strategies should be developed to reduce the prevalence and risk factors of chronic kidney disease among diabetes patients.

## 1. Background

Chronic kidney disease (CKD) is defined as structural/functional abnormalities of the kidney or decreased GFR <60 ml/min/1.73 m^2^ for 3 months [1]. It is an emerging global public health problem [2]. Globally, in 2017, there were 697.5 million cases of all-stage CKD, and 1.2 million people died each year due to high economic cost treatment [3]. In addition, it has been estimated that, by the year 2030, approximately 2.3–7.1 million adults have died prematurely from lack of access to renal replacement therapy [4]. The burden of CKD has been increasing, particularly in Oceania, sub-Saharan Africa, and Latin America [3]. Hence, developing countries have insufficient resources to address the CKD epidemic and its serious long-term complications. It has a significant economic burden, with treatment costs far exceeding preventive costs. For instance, a study performed in London revealed that the total yearly cost for the treatment of CKD was £1.44 to £1.45 billion, and more than half spent on renal replacement therapy, which was provided for 2% of the CKD population [5].

Although there is still uncertainty about the root cause of CKD, studies suggest that numerous risk factors are responsible for CKD, such as obesity [6–11], old age [7–9, 11–14], hypertension [3, 7, 9–13, 15–17], diabetes mellitus [3, 7, 8, 10–12, 16, 18], male gender [12, 17], hyperlipidemia [7], use of nephrotoxic medications [7], family history of kidney disease [9, 11, 13], smoking [19], heavy drinking [19], HIV infection [13], electrolyte and acid-base disturbances [13], low-income occupation, use of traditional medication, and low hemoglobin [11]. Early detection and treatment of possible risk factors are readily available and often inexpensive. Patients with CKD often suffer from an increased risk of cardiovascular mortality [20], ischemic heart disease [21], stroke [22], peripheral vascular disease [23], gout [24], depression and anxiety [25, 26], and reductions in patients' quality of life and markedly increases health care costs [27]. Patients with CKD may eventually progress to end-stage kidney disease (ESKD), which is associated with a high burden of disease and significant costs of treatment [28].

Large differences have been reported in terms of the prevalence of CKD based on available studies. For instance, the prevalence of CKD among diabetes patients is 38.5% in Palestine [29], 34.7% in Morocco [30], 18.2% in Ethiopia [31], and 24.6% in South Africa [32]. Evidence suggests that early detection and treatment of diabetes, hypertension, and other chronic diseases can improve renal outcomes and slow or prevent the progression of CKD [33]. Despite the availability of such interventions, the burden of CKD and its related risk factors remain understudied in developing countries. This would be due to low awareness among the public, health care workers, and government and other funders and may lead to the false perception that CKD is not an important problem in sub-Saharan Africa [34].

Although different primary studies have shown that the burden of CKD among diabetes patients is high and treatment options are expensive, their results have demonstrated substantial variation regarding its prevalence in Ethiopia. Given this, there is a strong imperative to fully understand the burden of CKD in the region. Therefore, this study aimed to estimate the pooled prevalence of CKD and its associated factors among diabetes patients in Ethiopia. This finding provides a scientific basis for a better understanding of the burden of CKD among diabetes mellitus patients and helps to design appropriate preventive strategies.

## 2. Methods

### 2.1. Data Source and Search Strategy

We conducted this systematic review and meta-analysis according to the protocol registered in PROSPERO (CRD42020204239), available at: https://www.crd.york.ac.uk/prospero/display_record.php?ID=CRD42020204239. The Preferred Reporting Items for Systematic Review and Meta-Analysis statement (PRISMA) guideline was used to report the pooled prevalence of CKD in patients with DM [35]. The literature was searched using PubMed, Scopus, Google Scholar, African Journals Online, and Wiley Online Library to identify published reports of kidney disease among diabetes patients in Ethiopia up to May 18, 2020. A manual search was performed for grey literature available on local university shelves and institutional repositories. Moreover, the reference lists of all retrieved articles were conducted to identify additional relevant research to minimize publication bias to possible levels. The search was restricted to full texts, free articles, human studies, and English language publications. Endnote X 8.1 reference manager software was used to search, collect, organize search outcomes, and remove duplicate articles. During the search, medical subheading (MeSH) as well as plain text was used for the following keywords: “chronic kidney disease,” “diabetic nephropathy,” “chronic renal failure,” “renal impairment,” “proteinuria,” “end-stage kidney/renal disease,” “renal insufficiency,” “diabetes mellitus,” “type 2 diabetes mellitus,” “type 1 diabetes mellitus,” “insulin dependent diabetes,” “non-insulin dependent diabetes”, and “Ethiopia”. We have followed the search protocol described in the previous publication [36], and we also used Boolean operators such as “AND” and “OR” which were used to combine search terms (Table 1).

### 2.2. Eligibility Criteria

Inclusion criteria for this study were as follows: (1) observational studies including cross-sectional studies, cohort studies (retrospective and prospective), and case-control studies that report a prevalence of CKD among diabetes patients in adults from Ethiopian were eligible for inclusion; (2) articles published in peer-reviewed journals or grey literature; and (3) articles published in English from inception to May 18, 2020. Furthermore, if different diagnostic criteria of CKD were found in a single study, our first choice was the Chronic Kidney Disease Epidemiology Collaboration (CKD-EPI), our second choice considered the Modification of Diet in Renal Disease (MDRD) study equation, and lastly, the Cockcroft–Gault formula in the main analyses. We excluded studies if (1) they were not fully accessible; (2) they possessed a poor quality score as per the stated criteria; (3) case series, letters, comments, and editorials; and/or (4) failed to measure the desired outcome (i.e., chronic kidney disease).

### 2.3. Outcome of Interest

The main outcome of interest was the prevalence of chronic kidney disease reported in the original paper both as a percentage and as the number of CKD cases (*n*)/total number of participants (*N*). CKD is defined as proteinuria, a creatinine clearance of less than 60 mL per min by the Cockcroft–Gault formula [37], or an estimated glomerular filtration rate (GFR) of less than 60 mL per min per 1.73 m^2^ by the Modification of Diet in Renal Disease (MDRD) equation [38] and Chronic Kidney Disease Epidemiology Collaboration (CKD-EPI) prediction equation [39].

### 2.4. Study Selection

Following the search, all identified citations were uploaded into EndNote version 8.1, and duplicates were removed. Titles and abstracts were then screened by two reviewers (WSS and YAA) for assessment against the inclusion criteria for the review. The full text of selected citations was assessed in detail against the inclusion criteria by two reviewers (TYA and YAA). Reasons for exclusion of full-text studies that did not meet the inclusion criteria were recorded and reported in the systematic review. Any disagreements that arose between the reviewers at each stage of the study selection process were resolved through discussion.

### 2.5. Data Extraction and Quality Assessment

After identifying articles for inclusion, two authors (WSS and TYA) performed data extraction. The Joanna Briggs Institute (JBI) tool was used for the data extraction [40]. For each included study, the following data were extracted: first/corresponding author, publication year, region, study design, sample size, data collection period, sampling technique, definition of kidney disease (microalbuminuria, albuminuria, macroalbuminuria, and estimated GFR (eGFR) decline), GFR equation/formula used, prevalence of CKD with its 95% confidence interval (CI), and associated factors. After data extraction, the third author (YAA) crosschecked both of the tables to ensure consistency. Any dispute that arose during data extraction was resolved by group consensus. The methodological quality of each included study was assessed using the Newcastle–Ottawa scale (NOS) [41]. This tool includes items that assess representativeness, response rate, the method of assessing outcomes, comparability of the subject, and the appropriateness of the statistical test used to analyze the data. Studies were included in the analysis if they scored ≥5 out of 10 points in three domains of ten modified NOS components for observational studies [42]. Furthermore, quality assurance checks were performed by two authors (YAA and WSS). Any controversy related to each article was collectively resolved by all authors, giving their opinion and the final decision made by consensus (supplementary file 1).

### 2.6. Assessment of Risk of Bias in Included Studies

An assessment of the risk of bias was conducted on all included studies developed by Hoy et al. [43] to assess the external and internal validity of nonrandomized studies in meta-analyses. The Hoy score is marked out of ten, and studies were classified as “high risk of bias” (total score ≤4), “moderate risk of bias” (total score between 5 and 7), or “low risk of bias” (total score between 8 and 10) (supplementary file 2). Two authors carried out the risk of bias assessment of the included studies.

### 2.7. Heterogeneity and Publication Bias

Cochran's *Q* and the *I*^2^ statistics were employed to investigate heterogeneity between studies [44], which estimates the percentage of total variation across studies due to true between-study differences rather than chance, with *I*^2^ values of 25, 50, and 75% representing low, medium, and high heterogeneity, respectively. We explored sources of heterogeneity through subgroup analysis and metaregression analysis. Sensitivity analysis was also performed for the effect of each study on the overall prevalence. Publication bias was assessed by visually inspecting funnel plots and objectively using Egger's test [45].

### 2.8. Statistical Analysis

We used the DerSimonian–Laird random-effects models to generate the pooled prevalence of CKD due to the anticipation of substantial variations in CKD prevalence estimates across the included studies [46]. The pooled effect size (i.e., prevalence) with a 95% confidence interval (CI) was generated and presented using a forest plot. All statistical analyses were performed using STATA™ version 14 software [47]. All the results are reported using PRISMA checklist (supplementary file 3).

## 3. Result

### 3.1. Selection of the Studies

**Figure 1:**
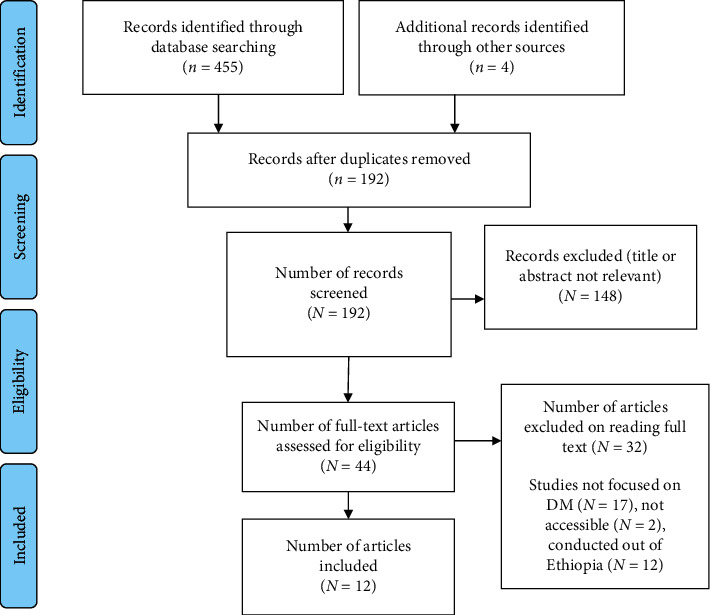
PRISMA flow diagram for selection of the included studies.

The initial search identified 490 articles, which were catalogued in citation management software (EndNote X 8.1). Of these, 455 studies were retrieved from PubMed (48), Scopus (20), Google Scholar (340), Wiley Online Library (19), and African Journals Online (59). On the other hand, the remaining 4 articles were found through manual search. Of them, 298 duplicate records were identified and removed. Following removal of duplicate studies, the titles and abstracts were evaluated, and 148 studies were excluded based on the prespecified inclusion criteria. Then, 44 studies were included for further assessment. After reviewing the full text, based on the predefined criteria and quality assessment, 12 articles were included for the final analysis (Figure 1).

### 3.2. Baseline Characteristics of the Study Participants

A total of 12 studies with 4,075 study participants were included in the review. Of these, 11 studies were used to estimate the pooled prevalence of CKD among diabetic patients. To estimate the pooled prevalence of CKD stages 1 to 5 among diabetes patients, seven studies [31, 48–53] were included. On the other hand, to estimate the pooled prevalence of CKD stages 3 to 5, ten studies [31, 48, 50–57] were included. Regarding the study design, the majority (75%) of the studies was cross-sectional. The subjects who participated in the studies were males and females aged ≥18 years. The number of study participants per study ranged from 189 to 700. The included studies applied various estimators of GFR; four of the studies [48, 55–57] used the CG formula in estimating GFR, four studies [31, 50, 51, 53] used the MDRD, and two studies [52, 54] used the CKD-EPI creatinine equation. The prevalence of CKD in patients with DM was obtained from various regions in Ethiopia; four studies were from Amhara [50, 51, 53], three from Addis Ababa [55–57], three from Oromia [48, 49, 52], one each from Tigray [58] and SNNPR [31]. With regard to the sampling technique, four studies [50, 51, 53, 56] used systematic random sampling, three studies [31, 57, 58] used simple random sampling, two studies [52, 54] used convenience sampling, and three studies [48, 49, 55] used the census sampling method. The quality score of the included studies was assessed based on the Newcastle–Ottawa quality score (Table 2).

### 3.3. Chronic Kidney Disease

**Figure 2:**
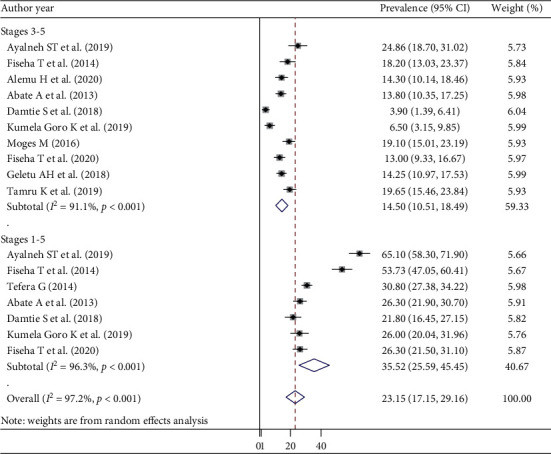
Pooled prevalence of chronic kidney disease (stages 1 to 5 and stages 3 to 5) among diabetes patients.

The current meta-analysis showed that the overall prevalence of CKD among diabetes patients was 35.52% (95% CI: 25.9–45.45, *I*^2^ = 96.3%) for CKD stages 1 to 5 and 14.5% (95% CI: 10.5–18.49, *I*^2^ = 91.1%) for CKD stages 3 to 5 (Figure 2).

### 3.4. Subgroup Analysis

To identify the source of heterogeneity across the included studies, subgroup analysis was deployed using regions, type of DM, study design, GFR equation/formula, and sampling technique. Based on the subgroup analysis results, the pooled prevalence of CKD stages 3 to 5 was 17.47% in studies conducted in Addis Ababa, 16.79% in patients with T2DM, 16.79% in studies conducted using cohort study design, 18.96% among studies using CG as GFR equation/formula, and 19.22% in studies with census sampling technique (Table 3).

### 3.5. Metaregression Analysis

To identify the source(s) of heterogeneity for CKD stages 1 to 5, metaregression analysis was undertaken by considering the year of publication, sample size, region, eGFR equation/formula, type of diabetes, study design, and sampling technique. However, our results showed that all covariates were not statistically significant for the presence of heterogeneity (Table 4).

### 3.6. Sensitivity

**Figure 3:**
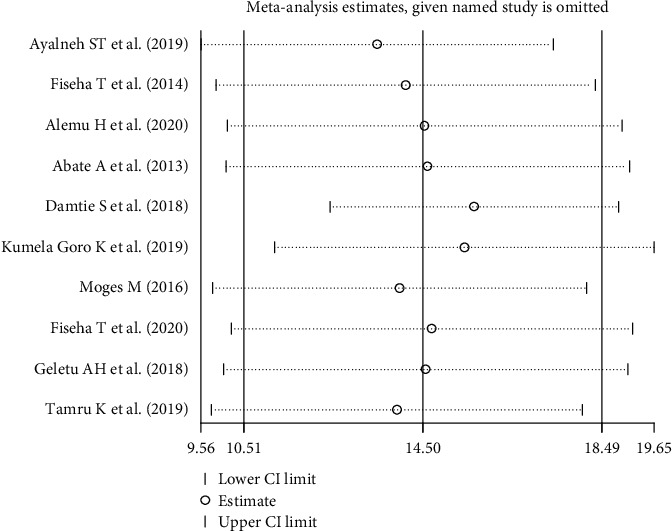
Results of the sensitivity analysis of the 10 studies.

In sensitivity analyses using the leave-one-out approach, excluding none of the studies had a significant effect on the pooled burden estimates and measures of heterogeneity within primary studies. Therefore, sensitivity analyses using the random-effects model revealed that no single study influenced the overall prevalence of CKD stages 3 to 5 among diabetic patients (Figure 3).

### 3.7. Publication Bias

**Figure 4:**
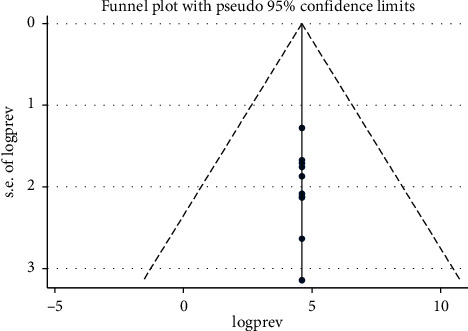
Funnel plot to test publication bias of the 10 studies.

As shown in Figure 4, the visual inspection of the funnel plot showed that there was no publication bias among the included studies, as illustrated by the symmetrical distribution of the funnel plot, when CKD stages 3 to 5 was evaluated. Likewise, the result of Egger's test was not statistically significant for the presence of publication bias (*P* = 0.806). On the other hand, when CKD stages 1 to 5 were analyzed, only 7 studies were included. Therefore, we did not display the funnel plot in this part, as the analyses are likely underpowered.

### 3.8. Factors Associated with Chronic Kidney Disease

Based on this meta-analysis, chronic kidney disease among diabetes patients in the Ethiopian context was associated with the duration of DM, female sex, age >60 years, family history of CKD, BMI ≥30 kg/m^2^, type 2 diabetes, FBG >150 mg/dl, HbA1c >7%, HDL ≥40 mg/dl, triglyceride >150 mg/dl, and systolic blood pressure >140 mmHg [31, 48, 51, 52, 54, 55, 57].

#### 3.8.1. Sociodemographic Factors

**Figure 5:**
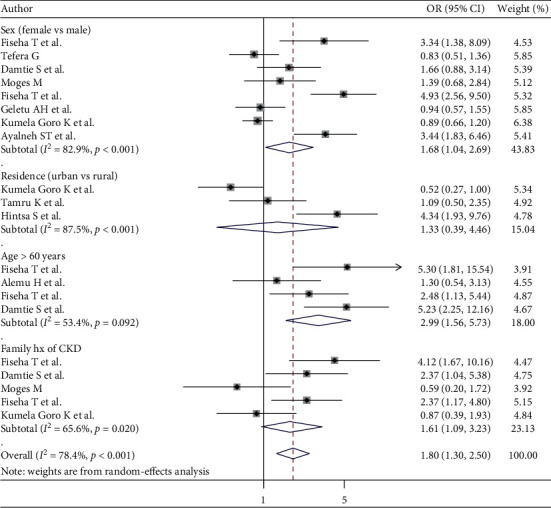
Sociodemographic factors associated with CKD in patients with DM.

The majority of the reports described the effects of age, sex, family history of CKD, and residence on CKD in patients with DM. Of these reports, only those that described the data in terms of the odds ratio, relative risk, and categorical variables were included. In the present analysis, the pooled effect of four studies [31, 51, 53, 54] showed that age greater than 60 years was statistically associated with CKD in patients with DM (OR = 2.99; 95% CI: 1.56, 5.73). The heterogeneity test (*I*^2^ = 53.4%) showed no significant evidence of variation across studies. Additionally, the pooled effect of eight studies [31, 48, 49, 51–53, 55, 56] showed that female sex had higher odds of CKD in patients with DM (OR = 1.68; 95% CI; 1.04, 2.69) than male sex. The heterogeneity test (*I*^2^ = 82.9%) showed significant evidence of variation across studies. Moreover, based on the pooled effects of five studies [31, 51–53, 56], those who had a family history of CKD were nearly two times more likely to develop CKD than patients without a family history of CKD (OR = 1.61; 95% CI: 1.09, 3.23, *I*^2^ = 65.6%). The details are presented in Figure 5.

#### 3.8.2. Behavioral Factors

**Figure 6:**
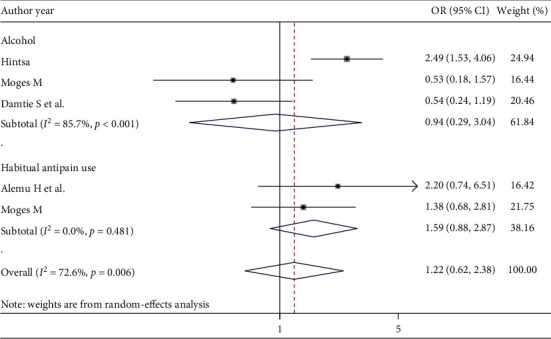
Behavioral factors associated with CKD among diabetes patients.

Most studies have reported that smoking, the use of traditional medicine, the habitual use of antipain drugs, and alcohol consumption are possible risk factors for CKD in patients with DM. Of these, only studies that reported data in terms of odds ratios, relative risks, and categorical variables were included. The pooled effects of two studies [54, 56] showed that a habitual use of antipain therapy was nearly two times more likely to develop CKD in patients with DM than in patients who did not habitually use antipain therapy (OR = 1.59; 95% CI: 0.88, 2.87, *I*^2^ = 0.0%), although this association was not statistically significant. In addition, clients who had current alcohol consumption practices had no significant difference in the development of CKD compared with those who could not consume alcohol (OR = 0.94; 95% CI: 0.29, 3.04, *I*^2^ = 85.7%) (Figure 6).

#### 3.8.3. Clinical Factors

**Figure 7:**
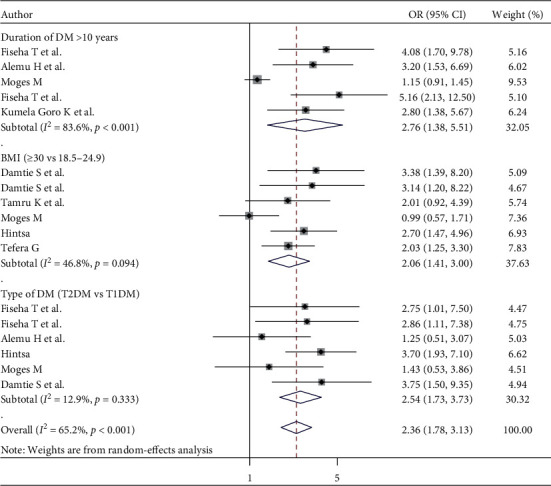
The effect of duration, BMI, and type of diabetes on CKD in patients with DM.

The pooled effects of five studies [31, 52–54, 56] showed that the duration of diabetes for >10 years was statistically associated with CKD in patients with DM (OR = 2.76; 95% CI: 1.38, 5.51). The heterogeneity test (*I*^2^ = 83.6%) shows moderate evidence of variation across studies. Additionally, patients with BMI ≥30 kg/m^2^ were 2.51 times more likely to develop CKD than those with BMI 18.5–24.9 kg/m^2^ (OR = 2.06; 95% CI: 1.41, 3.00, *I*^2^ = 46.8%) (Figure 7). Moreover, the pooled effects of six studies [31, 49, 51–53] showed that those patients who had fasting blood glucose >150 mg/dl were statistically associated with CKD in patients with DM (OR = 2.58; 95% CI: 1.79, 3.72). The heterogeneity test (*I*^2^ = 0.0%) showed no significant evidence of variation across studies (supplementary file 4). On the other hand, the current meta-analysis showed that patients with high-density lipoprotein greater than 40 mg/dl were 52% less likely to develop CKD than patients with high-density lipoprotein less than 40 mg/dl (OR = 0.48; 95% CI: 0.30, 0.77, *I*^2^ = 0.0%) (supplementary file 5).

#### 3.8.4. Comorbidity-Related Factors

**Figure 8:**
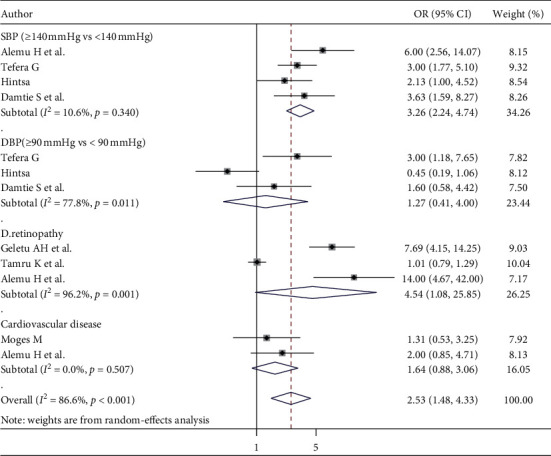
The effect of comorbidity-related factors on CKD inpatients with DM.

The pooled effects of four studies [49, 51, 54, 58] indicated that those patients who had systolic blood pressure >140 mmHg were 3.26 times more likely to develop CKD in patients with DM than SBP <140 mmHg (OR = 3.26; 95% CI: 2.24, 4.74, *I*^2^ = 10.6%). Additionally, nearly four patients who had diabetic retinopathy were more likely to develop CKD in patients with DM than in patients who did not have diabetic retinopathy (OR = 4.54; 95% CI: 1.08, 25.85, *I*^2^ = 96.2%). Moreover, in our study, there was no statistically significant association between CKD and those who had cardiovascular disease (OR = 1.64; 95% CI: 0.88, 3.06, *I*^2^ = 0.0%) (Figure 8).

## 4. Discussion

In the present review, the pooled prevalence of CKD among diabetes patients in Ethiopia was estimated to be 35.52% for CKD stages 1 to 5 irrespective of the diagnostic criteria. Additionally, we found that 14.5% of diabetes patients have moderate or severe decreases in kidney function (i.e., CKD stages 3 to 5). The overall estimated prevalence of CKD stages 1 to 5 among diabetes patients found in our review was higher than that reported in the burden of CKD on the African continent (32.6%) [59] and other systematic reviews conducted in Africa (24.7%) [60]. The above disparities could be a systematic review conducted on the African continent, and all studies were low quality except for four with medium quality [60]. Moreover, differences in sample size, demographics, presence of comorbidities, difference definition used to determine renal failure, and clinical characteristics may contribute to such variation [61]. Furthermore, our findings suggest that the prevalence of CKD is substantially higher in people receiving care for DM, which further substantiates the call to integrate DM treatment with the care of other noncommunicable diseases.

Based on the eGFR estimation equation (CKD-EPI, CG, and MDRD) criteria used across the included studies, the highest prevalence of CKD stages 3 to 5 among diabetes patients (18.96% (95% CI: 14.93–22.99)) was reported across studies using the CG diagnostic criteria, and the lowest prevalence (10.3% (95% CI: 2.66, 17.94)) of CKD was reported across studies using the CKD-EPI definition as a diagnostic criterion. Similar findings in the variation of CKD prevalence per diagnostic criteria were also reported in a study conducted in different countries [59, 60]. Though the CG equation showed a prevalence that was higher than the prevalence obtained in our findings using MDRD or CKD-EPI equations, the validity of those methods in the Ethiopian context remains to be established [62].

Risk factor association was assessed in 11 of the 12 studies based on the pooled analysis of the adjusted odds ratio of studies. Systolic blood hypertension, high density lipoprotein >40 mg/dl, BMI ≥30 kg/m^2^, HbA1c >7%, fasting blood glucose >150 mg/dl, triglyceride >150 mg/dl, female sex, diabetic retinopathy, family history of CKD, age >60 years, and duration of diabetes >10 years were associated with CKD in diabetic patients.

Although most of the studies to date reporting on sex differences in CKD associated with DM show either the male or female sex being a risk for CKD, a couple of studies have reported no effect of sex on CKD risk or progression [63, 64]. However, the results of a recent study showed that female sex is at a higher risk of CKD in patients with DM than in men. This finding is consistent with previous research conducted in Bethesda [65] and Nigeria [11]. However, studies from Saudi Arabia [66] and Italy [67] showed that men with type 2 diabetes have a higher prevalence of diabetes nephropathy. The variation could be the fact that men have more muscle mass than women, and the differences in hormone metabolism and glomerular structure are assumed to play a role in the differences in prevalence of CKD observed between male and female genders [68]. Additionally, in the setting of diabetes, it is generally believed that female sex as a protective factor is lost even before menopause [69], possibly due to the imbalance in sex hormone levels and activity.

The present study revealed that age>60 years leads to a three times greater likelihood of developing CKD. This finding is in support of previous studies conducted in Nigeria [11] and a systematic review of diabetic nephropathy in Africa [70]. Therefore, our data remind clinicians to strengthen diabetes care programs to meet the rising challenge of CKD. Additionally, screening among such a highly selected population may help identify those that would most benefit from modifiable factors, including lifestyle changes, associated with the progression of diabetic CKD, especially in early stages [71]. The results of this study showed that the increased duration of the disease had a statistically significant effect on CKD in patients with DM. This finding is consistent with previous research conducted in Italy [67], Africa [70], and the UK [72]. This suggests that optimization of the delivery of diabetes care prior to the development of CKD may lead to a reduction in the incidence and progression of early diabetic CKD.

In accordance with previous longitudinal and meta-analysis studies [66, 67, 70, 73], we found that BMI >30 kg/m^2^ was statistically associated with CKD incidence and progression in patients with DM, with a moderate degree of heterogeneity in the meta-analysis (*I*-squared value of 46.8%). Although the mechanisms that underlie the relationship between obesity and CKD are still poorly understood, some evidence has shown that excessive lipid deposition into the kidney as a result of obesity can also lead to the accumulation of toxic metabolites derived from fatty acid metabolism [74].

The current review shows that poor glycemic control (HbA1c >7%) is the most important risk factor for diabetic nephropathy. This was the same finding in different ethnic populations, as reported in Taiwanese [75], Saudi Arabia [66], and global meta-analysis studies [76]. In addition, the United Kingdom Prospective Diabetes Study (UKPDS) trial of patients with type 2 DM and preserved kidney function demonstrated that intensive glycemic control targeting an HbA1C level of <6–6.5% reduced the development and progression of diabetic nephropathy [77]. Moreover, additional studies should explore the physiopathological mechanism of HbA1C >7% that leads to renal complications.

Chronic kidney disease onset was also predicted by the typical atherogenic lipid profile. In the current review, high levels of triglycerides were directly associated with an increased probability of developing reduced eGFR, whereas HDL-c levels decreased the onset of CKD. This finding was consistent with previous reports in Italy [67] and Taiwan [78]. Accordingly, Penno et al. recently confirmed the independent association between hypertriglyceridemia and CKD among patients with type 2 diabetes mellitus in a cross-sectional study [79]. Therefore, our study verified that a stable HDL-C and a higher mean HDL-C are important protectors against the development of CKD in DM patients under a comprehensive diabetic care program.

The present study showed that systolic blood pressure greater than 140 mmHg was a strong predictor of CKD in patients with DM. The results are consistent with those of previous systematic reviews and meta-analyses [70, 72]. Evidence also supports that early treatment of hypertension is important in preventing cardiovascular disease and the progression of diabetic renal disease and retinopathy [80], and the benefit of tight blood pressure control may be as great or greater than strict glycemic control [81]. In addition, another meta-analysis study reported that diabetic patients may benefit more from intensive BP-lowering strategies to provide protection against kidney failure events in patients with diabetes [82, 83].

In the present review, patients with diabetic retinopathy increased the risk of CKD by 4.54 times compared with those with no diabetic retinopathy, with a high degree of heterogeneity in the meta-analysis (*I*-squared value of 96.2%). This finding is in agreement with studies conducted in Korea [84], Saudi Arabia [85], and Singapore [86]. A possible explanation might be that patients with DR and diabetic CKD are both microvascular complications that lead to extravasation and inflammation [87]. Therefore, clinicians should evaluate DR severity at the first visit and closely monitor renal function and albuminuria in subjects with severe DR.

This study has implications for clinical practice. Estimating the national burden of CKD among diabetes patients should likely be established as the initial step in kidney disease prevention whenever affordable and feasible. Providing guidance to enhance awareness of CKD among health care professionals and patients and the promotion of healthy lifestyles should be engrained in preventive programs. Moreover, it provides information about the burden and public health impact of renal failure in the county for possible attention during routine clinical patient care. Furthermore, identifying risk factors may help health care professionals treat DM patients with CKD during their clinical care.

This systematic review is not free from limitations. First, there was a large discrepancy in the definitions used to identify CKD and the methods of creatinine measurement. Second, it may lack national representativeness because no data were found from all regions of the country. Third, the majority of the included studies were cross-sectional study designs and cause-effect relationships; therefore, they cannot be reflected in this review.

## 5. Conclusion

This study revealed that the prevalence of CKD remains high among diabetes patients in Ethiopia based on the 11 research-based papers included in this study. Its prevalence varies across countries in the region, with the highest prevalence in Addis Ababa. This study found that a long duration of DM, age >60 years, diabetic retinopathy, female sex, family history of CKD, poor glycemic control, systolic blood pressure, BMI >30 kg/m^2^, and high density lipoprotein >40 mg/dl were significantly associated with an increased risk of CKD among diabetic patients. The findings provide a scientific basis for a further understanding of the risk factors of CKD in patients with DM and serve as a baseline for preventive strategies. Therefore, situation-based interventions and context-specific preventive strategies should be developed to reduce the prevalence and risk factors of CKD among diabetes patients.

## Figures and Tables

**Table 1:** PubMed search history.

Search	Search terms	Hits
1	Diabetes[tw] OR diabetes mellitus[tw] OR type 1 diabetes[tw] OR type 1 diabetes mellitus[tw] OR T1DM[tw] OR type 2 diabetes[tw] OR type 2 diabetes mellitus[tw] OR T2DM[tw]	623,574
2	Chronic kidney disease [tw] OR diabetic nephropathy[tw] OR chronic renal failure [tw] OR renal impairment [tw] OR proteinuria [tw] OR end-stage kidney/renal disease[tw] OR renal insufficiency [tw]	191,757
3	#1 and #2	168,020
4	Ethiopia[tw] OR ethio[tw]	18, 268
5	#3 and #4	127
6	Limits: studies done in humans, English language, and full text	48

**Table 2:** Baseline characteristics of the included studies.

Author publication	Pub year	Region	Health facility	Study design	Type of DM	Sample size	Prevalence (%)	Diagnostic criteria	Sampling technique	Study period	Quality score
Ayalneh et al. [48]	2019	Oromia	Asella Referral and Teaching Hospital	Cross-sectional	Type 1 & 2	189	24.86	Cockroft–gault	Census	September 2016 to December 2018	6
Fiseha et al. [31]	2014	SNNPR	Butajira Hospital	Cross-sectional	Type 1 & 2	214	18.2	MDRD	Simple random	September 1 to October 31, 2013	7
Alemu et al. [54]	2020	Amhara	University of Gondar Hospital	Cross-sectional	Type 1 & 2	272	14.3	CKD-EPI	Convenience	April 2 to July 31, 2018	6
Tefera [49]	2014	Oromia	Shakiso Health Center	Cross-sectional	Type 2	700	30.8	proteinuria	Census	July 2013 to 2014	7
Abate et al. [50]	2013	Amhara	Fenote Selam Hospital	Cross-sectional	Type 1 & 2	384	13.8	MDRD	Systematic random	February to April 2012	8
Damtie et al. [51]	2018	Amhara	University of Gonder hospital	Cross-sectional	Type 1 & 2	229	21.8	MDRD	Systematic random	February to April 2016	8
Kumela Goro et al. [52]	2019	Oromia	Jimma University Medical	Cross-sectional	Type 1 & 2	208	26	CKD-EPI	Convenience	Unspecified	6
Moges [56]	2016	Addis Ababa	Public hospitals of Addis Ababa	Cross-sectional	Type 1 & 2	355	19.1	Cockcroft–Gault	Systematic random	April 20 to May 12, 2016	7
Fiseha and Tamir [53]	2020	Amhara	Dessie Referral Hospital	Cross-sectional	Type 1 & 2	323	26.3	MDRD	Systematic random	February 1 to July 30, 2016	7
Geletu et al. [55]	2018	Addis Ababa	St. Paul's Hospital	Cohort study	Type 2	435	14.25	Cockcroft–Gault	Census	Unspecified	6
Tamiru et al. [57]	2019	Addis Ababa	Black Lion Specialized Hospital	Cohort study	Type 2	346	19.65	Cockcroft–Gault	Simple random	March 1 to April 28, 2019	8
Hintsa et al. [58]	2017	Tigray	Ayder Referral Hospital	Case control	Type 1 & 2	420	—	Not specified	Simple random	February 14 to May 8, 2016	7

MDRD: Modification of Diet in Renal Disease equation; CKD-EPI: Chronic Kidney Disease Epidemiology Collaboration prediction equation.

**Table 3:** The results of subgroup analysis by different categories of the studies.

Subgroup	Category	No. of studies	Sample size	Stages 3–5 Prevalence (95% CI)	*P* value	*I* ^2^ (%)
Region	Amhara Addis ababa Others	4 3 3	1,208 1,136 611	11.13 (5.47, 16.79) 17.47 (13.88, 21.05) 16.3 (5.13, 27.46)	<0.001 0.073 <0.001	91.1 61.8 93.8
Study design	Cohort Cross-sectional	2 9	781 2,874	16.79 (11.51, 22.07) 13.92 (9.23, 18.16)	0.047 <0.001	74.7 91.8
Type of DM	Type 2 Type 1 & 2	2 8	781 2,174	16.79 (11.51, 22.07) 13.92 (9.23, 18.61)	0.047 <0.001	74.7 91.8
GFR equation/formula	MDRD CKD-EPI CG	4 2 4	1,150 480 1,325	12.02 (5.62, 18.42) 10.3 (2.66, 17.94) 18.96 (14.93, 22.99)	<0.001 0.004 0.014	92.4 87.8 71.8
Sampling technique	Systematic random Simple random Convenience Census	4 2 2 3	1,291 560 480 1,324	12.34 (5.56, 19.11) 19.08 (15.82, 22.33) 10.3 (2.66, 17.94) 19.22 (8.84, 29.6)	<0.001 0.669 0.004 0.003	93.9 0.0 87.8 88.7

CG: Cockcroft–Gault; MDRD: Modification of Diet in Renal Disease equation; CKD-EPI: Chronic Kidney Disease Epidemiology Collaboration prediction equation.

**Table 4:** Metaregression analysis for the included studies to identify the source(s) of heterogeneity.

Variables	Category	Coef.	Std. error	*t* value	*P* value	(95% conf. interval)
Sample size	Total sample size	−0.0002	0.001	−0.14	0.895	(−0.004, 0.003)
Year of publication		0.069	0.303	0.23	0.824	(−0.607, 0.745)
Region	Amhara Addis Ababa Others (reference)	−0.011 0.015	2.851 2.297	−0.001 0.001	0.997 0.995	(−6.365, 6.341) (−5.104, 5.134)
Study design	Cross-sectional Cohort (reference)	0.8430	2.231	0.38	0.713	(−4.128, 5.814)
Type of DM	Type 2 Type 1 & 2 (reference)	−0.102	2.260	−0.05	0.965	(−5.139, 4.934)
GFR equation/formula	MDRD CKD-EPI CG (reference)	0.099 0.013	1.646 1.472	0.06 0.01	0.953 0.993	(−3.568, 3.767) (−3.266, 3.293)
Sampling technique	Systematic random Simple random Census Convenience (reference)	0.159 0.542 0.245	2.244 2.561 1.678	0.07 0.21 0.72	0.945 0.837 0.563	(−4.840, 5.159) (−5.166, 6.250) (−3.456, 4.951)

CG: Cockcroft–Gault; MDRD: Modification of Diet in Renal Disease equation; CKD-EPI: Chronic Kidney Disease Epidemiology Collaboration prediction equation.

## Data Availability

The data used to support the findings of this study are available within the article and its supplementary information files.

## Abbreviations

AOR: Adjusted odds ratio
CI: Confidence interval
CKD: Chronic kidney disease
CKD-EPI: Chronic Kidney Disease Epidemiology Collaboration prediction equation
DM: Diabetes mellitus
eGFR: Estimated glomerular filtration rate
MDRD: Modification of Diet in Renal Disease equation
NOS: Newcastle–Ottawa scale
PRISMA: Preferred Reporting Items for Systematic Reviews and Meta-Analyses.

## Supplementary Materials

Supplementary Materials Supplementary file 1: methodological quality assessment of the included studies was performed using the modified Newcastle–Ottawa Scale (NOS). Supplementary file 2: the risk of bias assessment tool for the included studies. Supplementary file 3: PRISMA checklist for meta-analysis of the prevalence of CKD among diabetes patients in Ethiopia. Supplementary file 4: the association of fasting blood glucose and glycemic control on CKD in patients with DM. Supplementary file 5: the effect of lipid profile on CKD in patients with DM.
