# Supplementary material for: Chronic Kidney Disease among Diabetes Patients in Ethiopia: A Systematic Review and Meta-Analysis
Source: Int J Nephrol. 2020 Oct 10;2020:8890331. doi: 10.1155/2020/8890331 (PMC7569456; doi:10.1155/2020/8890331)
Supplement: Supplementary Materials — Supplementary file 1: methodological quality assessment of the included studies was performed using the modified Newcastle–Ottawa Scale (NOS). Supplementary file 2: the risk of bias assessment tool for the included studies. Supplementary file 3: PRISMA checklist for meta-analysis of the prevalence of CKD among diabetes patients in Ethiopia. Supplementary file 4: the association of fasting blood glucose and glycemic control on CKD in patients with DM. Supplementary file 5: the effect of lipid profile on CKD in patients with DM. [file 8890331.f1.docx]

**Methodological quality assessment**

**Supplementary file 1:** Methodological quality assessment of included studies using modified Newcastle - Ottawa Scale (NOS)

| **Corresponding author**  **[reference]** |  | **Criteria** | | | | | | | | |  |
| --- | --- | --- | --- | --- | --- | --- | --- | --- | --- | --- | --- |
|  |  | **Selection** | | | | | **Comparability** | | **Outcome** | |  |
|  | **Study Design** | | **Representativeness of the sample** | **Sample size** | **Non –responders** | **Ascertainment of exposure/risk factor** | **The study controls for the most important factor** | **The study control for any additional factor** | **Assessment of the outcome** | **Statistical test** | **Quality score** |
| Ayalneh ST etal.[49] | Cross-sectional | | A* | **A*** | **B*** | **B*** | **-** | **-** | A* | **A*** | **6** |
| Fiseha T etal. [30] | Cross-sectional | | A* | **A*** | **B*** | **B*** | **B*** | **-** | A* | **A*** | **7** |
| Alemu H etal. [53] | Cross-sectional | | B* | **B*** | **B*** | **B*** | **-** | **-** | A* | **A*** | **6** |
| Tefera G.[55] | Cross-sectional | | B* | **A*** | **B*** | **A*** | **-** | **B*** | A* | **A*** | **7** |
| Abate A etal.[50] | Cross-sectional | | A* | **A*** | **A*** | **B*** | **B*** | **-** | A* | **A*** | **8** |
| Damtie S eatl.[51] | Cross-sectional | | A* | **A*** | **A*** | **B*** | **B*** | **-** | A* | **A*** | **8** |
| Kumela Goro K etal. [54] | Cross-sectional | | B* | **B*** | **B*** | **B*** | **-** | **B*** | A* | **A*** | **6** |
| Moges M.[47] | Cross-sectional | | A* | **A*** | **A*** | **A*** | **B*** | **-** | A* | **A*** | **7** |
| Fiseha T etal.[52] | Cross-sectional | | A* | **A*** | **B*** | **A*** | **A*** | **-** | A* | **A*** | **7** |
| Geletu AH etal [48] | Retrospecive cohort | | B* | **B*** | **B*** | **B*** | **-** | **B*** | A* | **A*** | **6** |
| Tamru K etal. [46] | Retrospective cohort | | A* | **A*** | **A*** | **A*** | **-** | **A*** | A** | **A*** | **8** |

*Note: from each item account point. (Accept the study for each study design based on total score of ≥50%)*

Selection: (Maximum 5 stars)
1) Representativeness of the sample: a) Truly representative of the average in the target population. * (all subjects or random sampling) .b) Somewhat representative of the average in the target population. * (nonrandom sampling) .c) Selected group of users.d) No description of the sampling strategy.
2) Sample size:a) Justified and satisfactory. *.b) Not justified.
3) Non-respondents: a) Comparability between respondents and non-respondents characteristics is
established, and the response rate is satisfactory. * .b) The response rate is unsatisfactory, or the comparability between respondents
and non-respondents is unsatisfactory. c) No description of the response rate or the characteristics of the responders and
the non-responders.
4) Ascertainment of the exposure (risk factor): a) validated measurement tool. ** .b) Non-validated measurement tool, but the tool is available or described.* c) No description of the measurement tool.
Comparability: (Maximum 2 stars)
1) The subjects in different outcome groups are comparable, based on the study design or analysis. Confounding factors are controlled. a) The study controls for the most important factor (select one). * b) The study control for any additional factor. *
Outcome: (Maximum 3 stars)
1) Assessment of the outcome: a) Independent blind assessment. **,b) Record linkage. **,c) Self report. *,d) No description.
2) Statistical test:a) The statistical test used to analyze the data is clearly described and appropriate, and the measurement of the association is presented, including confidence intervals and the probability level (p value). *,b) The statistical test is not appropriate, not described or incomplete

**Supplementary file 2:** The risk of bias assessment tool for the included studies.

| **Corresponding author**  **[reference]** | representation | Sampling | random selection | non-response bias | data collected | case definition | reliability &validity of tool | mode of data collection | length prevalence period | numerator & denominator | the overall risk of bias |
| --- | --- | --- | --- | --- | --- | --- | --- | --- | --- | --- | --- |
| Ayalneh ST etal.[49] | No | No | No | No | Yes | Yes | Yes | Yes | Yes | Yes | Moderate risk |
| Fiseha T etal. [30] | Yes | Yes | Yes | Yes | Yes | Yes | Yes | Yes | Yes | Yes | Low risk |
| Alemu H etal. [53] | No | No | No | No | Yes | Yes | Yes | Yes | Yes | Yes | Moderate risk |
| Tefera G.[55] | Yes | No | No | No | Yes | Yes | Yes | Yes | Yes | Yes | Moderate risk |
| Abate A etal.[50] | Yes | Yes | Yes | No | Yes | Yes | Yes | Yes | Yes | Yes | Low risk |
| Damtie S eatl.[51] | Yes | Yes | Yes | Yes | Yes | Yes | Yes | Yes | Yes | Yes | low risk |
| Kumela Goro K etal. [54] | No | No | No | No | Yes | Yes | Yes | Yes | Yes | Yes | Moderate risk |
| Moges M.[47] | Yes | Yes | Yes | No | Yes | Yes | Yes | Yes | No | Yes | Low risk |
| Fiseha T etal.[52] | Yes | Yes | Yes | Yes | Yes | Yes | Yes | Yes | Yes | No | Low risk |
| Geletu AH etal [48] | No | No | No | No | Yes | Yes | Yes | Yes | Yes | Yes | Moderate risk |
| Tamru K etal. [46] | Yes | Yes | Yes | Yes | Yes | Yes | Yes | Yes | Yes | Yes | Low risk |

- **Note**

Risk of bias assessment tool: Yes (low risk); No (high risk)

1. Representation: Was the study population a close representation of the national population?

2. Sampling: Was the sampling frame a true or close representation of the target population?

3. Random selection: Was some form of random selection used to select the sample OR was a census undertaken?

4. Non-response bias: Was the likelihood of non-response bias minimal?

5. Data collection: Were data collected directly from the subjects?

6. Case definition: Was an acceptable case definition used in the study?

7. Reliability and validity of study tool: Was the study instrument that measured the parameter of interest show to have reliability and validity?

8. Data collection: Was the same mode of data collection used for all subjects?

9. Prevalence period: Was the length of the prevalence period for the parameter of interest appropriate?

10. Numerators and denominators: Were the numerator(s) and denominator(s) for the parameter of interest appropriate?

The overall risk of bias scored based on the number of high risk of bias per study: low risk (≥8), moderate risk (5–7), and high risk (≤4)

Supplementary file 3. PRISMA checklist for meta-analysis of the prevalence of CKD among diabetes patients in Ethiopia

| **Section/topic** | **#** | | **Checklist item** | | **Reported on page #** |
| --- | --- | --- | --- | --- | --- |
| **TITLE** | | | | |  |
| Title | 1 | | Identify the report as a systematic review, meta-analysis, or both. | | 1 |
| **ABSTRACT** | | | | |  |
| Structured summary | 2 | | Provide a structured summary including, as applicable: background; objectives; data sources; study eligibility criteria, participants, and interventions; study appraisal and synthesis methods; results; limitations; conclusions and implications of key findings; systematic review registration number. | | 2 |
| **INTRODUCTION** | | | | |  |
| Rationale | 3 | | Describe the rationale for the review in the context of what is already known. | | 3-4 |
| Objectives | 4 | | Provide an explicit statement of questions being addressed with reference to participants, interventions, comparisons, outcomes, and study design (PICOS). | | N/A |
| **METHODS** | | | | |  |
| Protocol and registration | 5 | | Indicate if a review protocol exists, if and where it can be accessed (e.g., Web address), and, if available, provide registration information including registration number. | | In progress #4 |
| Eligibility criteria | 6 | | Specify study characteristics (e.g., PICOS, length of follow-up) and report characteristics (e.g., years considered, language, publication status) used as criteria for eligibility, giving rationale. | | 5 |
| Information sources | 7 | | Describe all information sources (e.g., databases with dates of coverage, contact with study authors to identify additional studies) in the search and date last searched. | | 4 |
| Search | 8 | | Present full electronic search strategy for at least one database, including any limits used, such that it could be repeated. | | 4-5 |
| Study selection | 9 | | State the process for selecting studies (i.e., screening, eligibility, included in systematic review, and, if applicable, included in the meta-analysis). | | 6 |
| Data collection process | 10 | | Describe method of data extraction from reports (e.g., piloted forms, independently, in duplicate) and any processes for obtaining and confirming data from investigators. | | 6 |
| Data items | 11 | | List and define all variables for which data were sought (e.g., PICOS, funding sources) and any assumptions and simplifications made. | | N/A |
| Risk of bias in individual studies | 12 | | Describe methods used for assessing risk of bias of individual studies (including specification of whether this was done at the study or outcome level), and how this information is to be used in any data synthesis. | | 7 |
| Summary measures | 13 | | State the principal summary measures (e.g., risk ratio, difference in means). | | 7 |
| Synthesis of results | 14 | | Describe the methods of handling data and combining results of studies, if done, including measures of consistency (e.g., I^2^) for each meta-analysis. | | 7 |
| **Section/topic** | | **#** | | **Checklist item** | **Reported on page #** |
| Risk of bias across studies | | 15 | | Specify any assessment of risk of bias that may affect the cumulative evidence (e.g., publication bias, selective reporting within studies). | 7 |
| Additional analyses | | 16 | | Describe methods of additional analyses (e.g., sensitivity or subgroup analyses, meta-regression), if done, indicating which were pre-specified. | 7 |
| **RESULTS** | | | | |  |
| Study selection | | 17 | | Give numbers of studies screened, assessed for eligibility, and included in the review, with reasons for exclusions at each stage, ideally with a flow diagram. | 7-8 |
| Study characteristics | | 18 | | For each study, present characteristics for which data were extracted (e.g., study size, PICOS, follow-up period) and provide the citations. | 8-9 |
| Risk of bias within studies | | 19 | | Present data on risk of bias of each study and, if available, any outcome level assessment (see item 12). | N/A |
| Results of individual studies | | 20 | | For all outcomes considered (benefits or harms), present, for each study: (a) simple summary data for each intervention group (b) effect estimates and confidence intervals, ideally with a forest plot. | N/A |
| Synthesis of results | | 21 | | Present results of each meta-analysis done, including confidence intervals and measures of consistency. | 11 |
| Risk of bias across studies | | 22 | | Present results of any assessment of risk of bias across studies (see Item 15). | 14 |
| Additional analysis | | 23 | | Give results of additional analyses, if done (e.g., sensitivity or subgroup analyses, meta-regression [see Item 16]). | 12-13 |
| **DISCUSSION** | | | | |  |
| Summary of evidence | | 24 | | Summarize the main findings including the strength of evidence for each main outcome; consider their relevance to key groups (e.g., healthcare providers, users, and policy makers). | 22-25 |
| Limitations | | 25 | | Discuss limitations at study and outcome level (e.g., risk of bias), and at review-level (e.g., incomplete retrieval of identified research, reporting bias). | 26 |
| Conclusions | | 26 | | Provide a general interpretation of the results in the context of other evidence, and implications for future research. | 26 |
| **FUNDING** | | | | |  |
| Funding | | 27 | | Describe sources of funding for the systematic review and other support (e.g., supply of data); role of funders for the systematic review. | No fund |

**Supplementary file 4.** The association of fasting blood glucose and glycemic control on CKD in patients with DM.

NOTE: Weights are from random effects analysis

.

.

Overall (I-squared = 17.0%, p = 0.300)

Hintsa

Author

Fiseha T etal

Tefera G

Tamru K etal

Kumela Goro K etal

FBG**(>150mg/dl vs ≤ 150mg/dl**)

Damtie S eatl

Fiseha T etal

HbA1c(**poor vs good**)

Subtotal (I-squared = 0.0%, p = 0.415)

Subtotal (I-squared = 39.3%, p = 0.199)

2.24 (1.72, 2.92)

2.71 (1.49, 4.94)

OR (95% CI)

2.58 (1.19, 5.60)

1.80 (0.59, 5.45)

1.74 (1.27, 2.38)

3.70 (1.73, 7.89)

1.75 (0.91, 3.37)

4.65 (1.69, 12.78)

2.58 (1.79, 3.72)

2.01 (1.34, 3.02)

100.00

15.69

Weight

10.19

5.33

38.24

10.61

%

13.61

6.32

46.06

53.94

2.24 (1.72, 2.92)

2.71 (1.49, 4.94)

OR (95% CI)

2.58 (1.19, 5.60)

1.80 (0.59, 5.45)

1.74 (1.27, 2.38)

3.70 (1.73, 7.89)

1.75 (0.91, 3.37)

4.65 (1.69, 12.78)

2.58 (1.79, 3.72)

2.01 (1.34, 3.02)

100.00

15.69

Weight

10.19

5.33

38.24

10.61

%

13.61

6.32

46.06

53.94

1

1

5

**Supplementary file 5.** The effect of lipid profile on CKD in patients with DM

NOTE: Weights are from random effects analysis

.

.

.

.

Overall (I-squared = 58.1%, p = 0.019)

LDL(≥100mg/dl vs <100mg/dl)

Tamru K etal

Triglyceride(≥150mg/dl vs <150mg/dl)

Tamru K etal

Total cholesterol(≥200mg/dl vs <200mg/dl)

Geletu AH etal

Author

Tamru K etal

Subtotal (I-squared = 0.0%, p = 0.654)

Subtotal (I-squared = 36.0%, p = 0.211)

Geletu AH etal

Tamru K etal

HDL(≥40mg/dl vs <40mg/dl)

Geletu AH etal

Subtotal (I-squared = 0.0%, p = 0.438)

Geletu AH etal

Subtotal (I-squared = 0.0%, p = 0.849)

1.02 (0.75, 1.40)

1.61 (0.91, 2.86)

0.37 (0.16, 0.84)

1.35 (0.81, 2.25)

OR (95% CI)

1.01 (0.56, 1.84)

1.46 (1.01, 2.14)

1.28 (0.79, 2.08)

1.02 (0.61, 1.71)

1.67 (0.94, 2.96)

0.55 (0.31, 0.97)

0.48 (0.30, 0.77)

1.09 (0.66, 1.81)

1.06 (0.72, 1.56)

100.00

12.56

8.71

13.81

Weight

12.15

26.37

26.27

13.66

12.60

12.66

21.38

13.84

25.99

%

1.02 (0.75, 1.40)

1.61 (0.91, 2.86)

0.37 (0.16, 0.84)

1.35 (0.81, 2.25)

OR (95% CI)

1.01 (0.56, 1.84)

1.28 (0.79, 2.08)

1.02 (0.61, 1.71)

1.67 (0.94, 2.96)

0.55 (0.31, 0.97)

0.48 (0.30, 0.77)

1.09 (0.66, 1.81)

1.06 (0.72, 1.56)

100.00

12.56

8.71

13.81

Weight

12.15

26.37

26.27

13.66

12.60

12.66

21.38

13.84

25.99

%

1

1

5
